# Recurrence of Cervical Lymph Node Metastasis From an Unknown Primary Site: A Report of a Case Treated Using a Multidisciplinary Approach

**DOI:** 10.7759/cureus.58537

**Published:** 2024-04-18

**Authors:** Nikhar Wadhwani, Nitin Bhola, Rajanikanth Kambala, Chetan Gupta

**Affiliations:** 1 Oral and Maxillofacial Surgery, Sharad Pawar Dental College and Hospital, Datta Meghe Institute of Higher Education and Research, Wardha, IND

**Keywords:** head and neck cancer surgery, neoplasm recurrence, carcinoma of unknown primary (cup), oncology and critical care, cervical lymphadenopathy

## Abstract

Metastatic cervical carcinoma from an unknown primary source poses a diagnostic and therapeutic challenge, as it involves the spread of cancer to the neck lymph nodes without a discernible primary tumor despite thorough investigation. While the diagnosis and treatment of this uncommon condition lack definitive evidence, a review of existing literature offers some clinical guidance. A comprehensive diagnostic evaluation, which includes multiple imaging and endoscopic studies, is essential. Surgery is preferred whenever feasible due to its ability to offer more precise staging. This treatment entails an excisional biopsy, neck dissection, and tonsillectomy, but advanced cases necessitate a combination of treatments.

This case report underscores this complexity, where, despite radical neck dissection on the affected side, recurrence manifested after two months with no discernible primary site. We emphasize the urgency for continued research and innovative approaches to enhance the diagnosis and management of metastatic cervical carcinoma from an unknown primary source.

## Introduction

Carcinoma of unknown primary origin pertains to cases where metastatic cancer is detected, yet the primary site remains unidentified despite thorough evaluation. These constitute 3-5% of all head and neck malignancies [1]. Efforts are directed toward identifying and removing the primary tumor, as this information is crucial for tailoring appropriate treatment. The management of cervical lymphadenopathy from occult primary tumors typically requires a collaborative effort from various medical specialists, utilizing advanced imaging and molecular profiling to enhance diagnostic accuracy. Along with malignancies arising in the oral cavity, cervical lymph node metastasis often arises from malignancies originating within Waldeyer's ring, encompassing lymphoid tissue in the nasopharynx, tonsil, and base of the tongue [2]. Through bimanual palpation, clinicians can detect abnormalities in the tonsillar fossae and base of the tongue, guiding potential biopsy procedures for further diagnosis. The predominant histological type is squamous cell carcinoma, representing 75-90% of cases, followed by undifferentiated carcinoma and adenocarcinoma [3]. We report the management of a case of cervical lymphadenopathy in which, despite exhaustive investigations, the primary tumor could not be found. The patient was treated for carcinoma of unknown primary origin by radial neck dissection of the affected side.

## Case presentation

**Figure 1:**
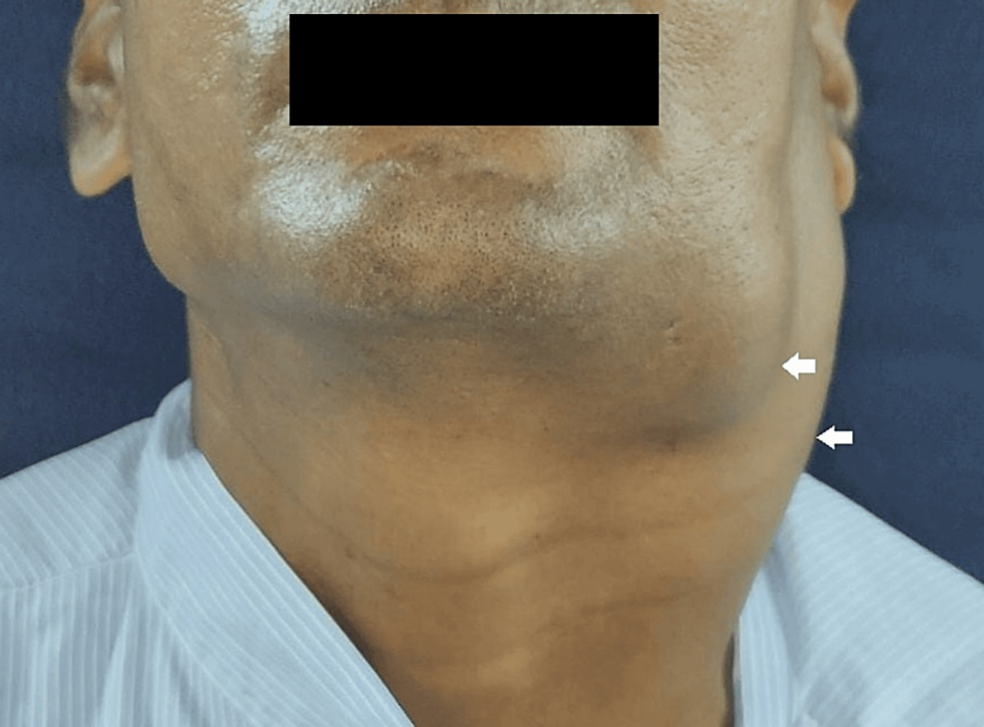
Multilobulated swelling present on the left side of the neck

A 46-year-old male reported to our facility with the complaint of a slow-growing swelling over the left side of his neck for two months, which was initially small in size and gradually increased to a size of approximately 8 x 7 cm. The patient presented with a history of pain persisting for the past 10 days. The pain had a gradual onset, was continuous, characterized by a dull aching sensation, and was localized in nature. Notably, the pain worsened during mastication and spontaneously subsided over time. He had no relevant previous medical history, including malignancies. He reported a history of tobacco consumption of three to four times per day for approximately seven years. External findings of the swelling revealed a multilobulated mass, seen in Figure 1, extending from the preauricular region to the level of the cricoid cartilage over the left side. The swelling was smooth, with no signs of erythema on the overlying skin, but on palpation, it was found to be tender and fixed. An intraoral examination showed no ulcerative growth or any dysplastic changes, although fibrous bands were palpable bilaterally over the buccal mucosa. There was no restriction in the movements of the tongue, nor was there any account of a change in voice.

**Figure 2:**
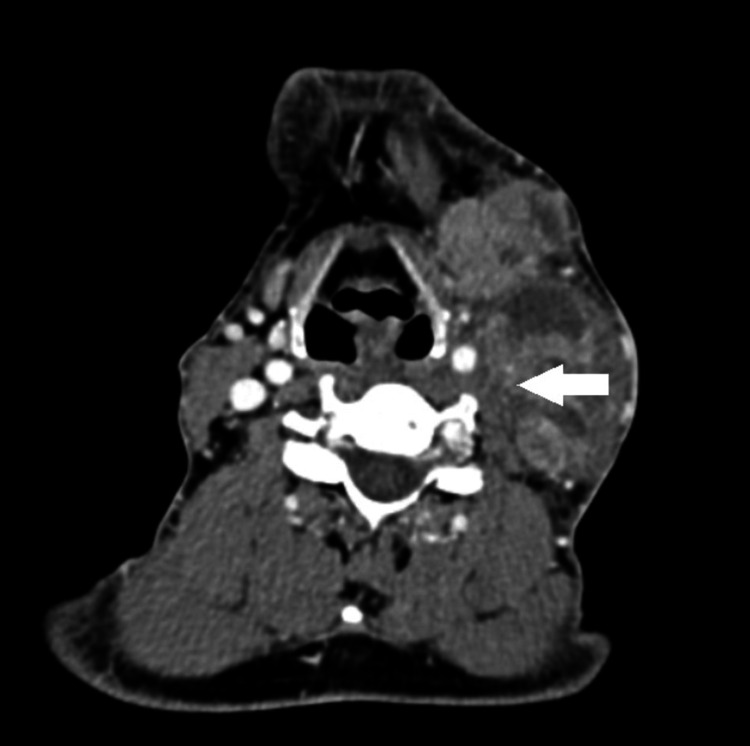
Computed tomography image of the neck (axial view) Computed tomography image of the neck, axial cut in venous phase showing encasement of the internal jugular vein.

After the oral cavity was examined, with futile results, a diagnostic endoscopy of the nose and upper aerodigestive tract was performed to evaluate the presence of any suspicious growth, but it did not report any lesions. In imaging studies, first a craniocervical computed tomography (CT) scan was advised to assess for any mass. It was suggestive of multiple metastatic lymph nodes involving the submental, left submandibular, upper, middle, and lower jugulodiagastric regions, the largest of which was 3.5 x 3.6 cm. There was a mass effect in the form of compression of the left jugular vein, as seen in Figure 2.

**Figure 3:**
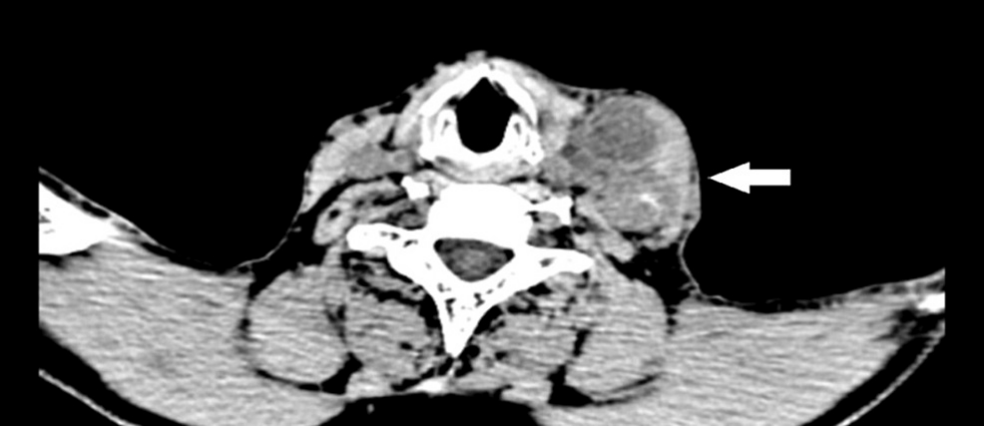
Computed tomography image of the neck (axial section) showing the extent of the lesion

The chain of involved lymph nodes can be seen extending up to the seventh cervical vertebra in Figure 3. 

As a lesion was not evident in the craniocervical CT scan, a positron emission tomography (PET) scan was done, which showed intense fludeoxyglucose (FDG) avidity heterogeneously enhancing large conglomerated left-sided cervical nodal mass extending from left cervical IB to left cervical level IV nodal stations, measuring 81 x 42 x 73 mm. The lesion was seen abutting the left submandibular gland and completely encasing the left internal jugular vein. It was also closely abutting the left common carotid artery and its branches. No suspicious lesion was seen in the brain, thorax, liver, spleen, or adrenal glands. No remarkably enlarged or hypermetabolic abdominal, retroperitoneal, pelvic, or inguinal lymphadenopathy was noted. A fine needle aspiration cytology of the mass over the left cervical region was done, which was suggestive of deposits of squamous cell carcinoma with granulomatous reactions.

Treatment and progression

**Figure 4:**
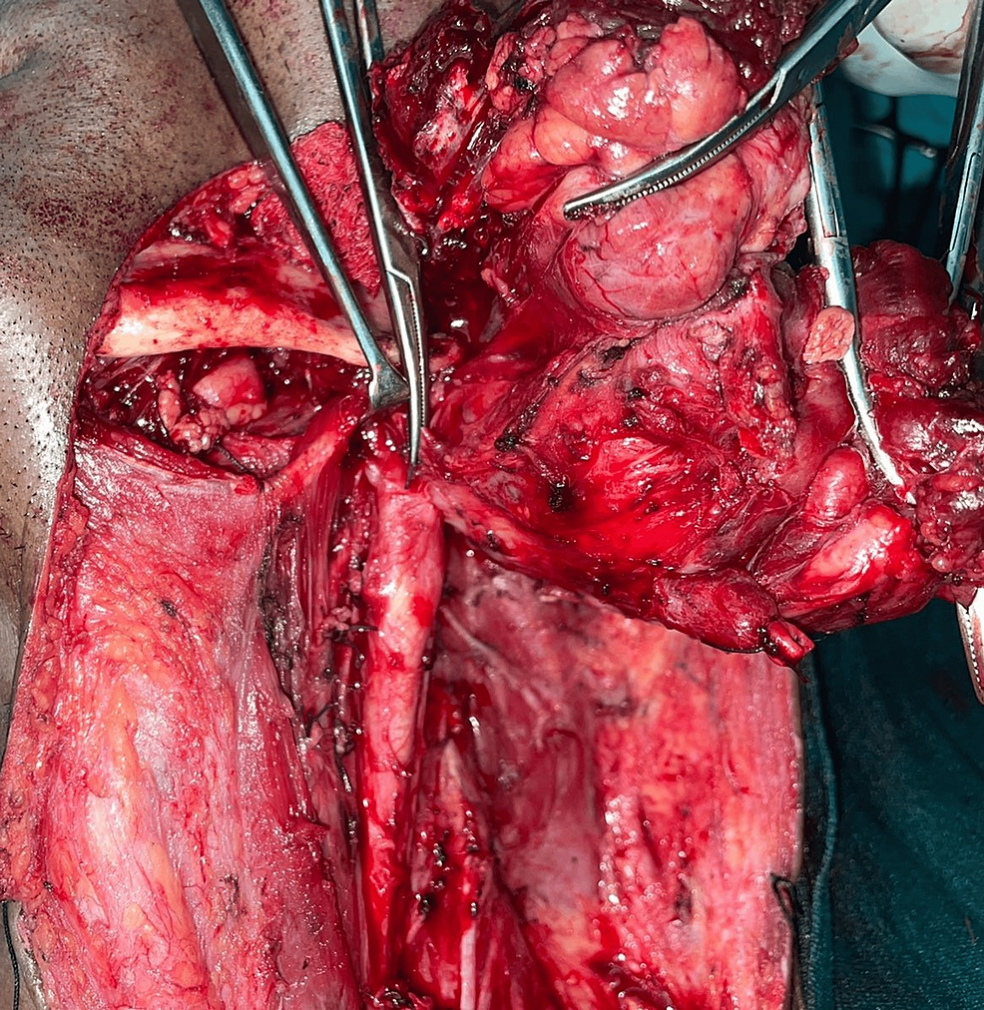
Radical neck dissection of the left side

Despite an extensive diagnostic workup including a PET-CT, craniocervical CT, nasal cavity examination, and oral cavity examination, no primary tumor was found in the craniocervical or any other region. The cervical tumor was thus diagnosed as a carcinoma of unknown primary. Radical surgery was judged difficult as the tumor was accompanied by the encasement of an internal jugular vein and closely abutting the left common carotid artery. After pre-anaesthetic clearance, the patient underwent radical neck dissection of the left side under general anesthesia, sacrificing lymph nodes from Level I to Level V, the submandibular gland, the internal jugular vein, the sternocleidomastoid muscle, and the spinal accessory nerve of the left side, as seen in Figure 4.

**Figure 5:**
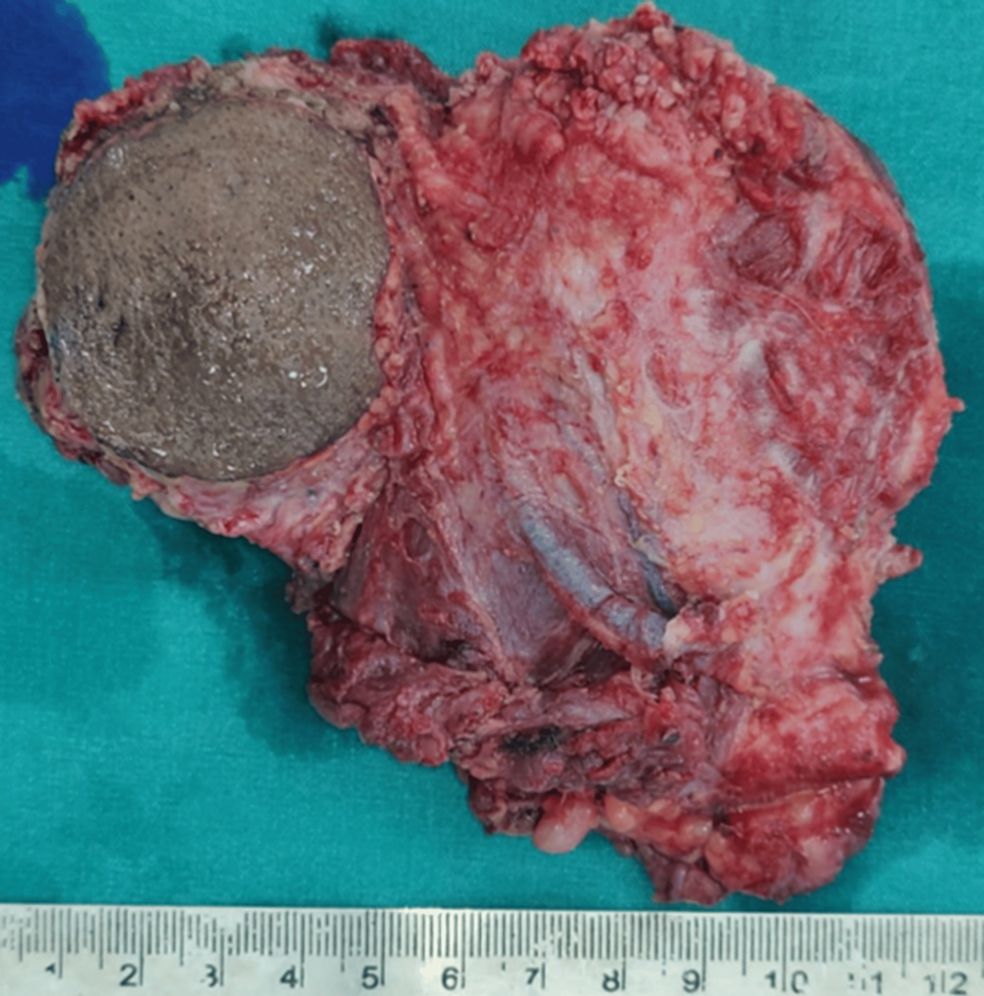
The resected tumour mass

The resected tumor mass can be seen in Figure 5. 

**Figure 6:**
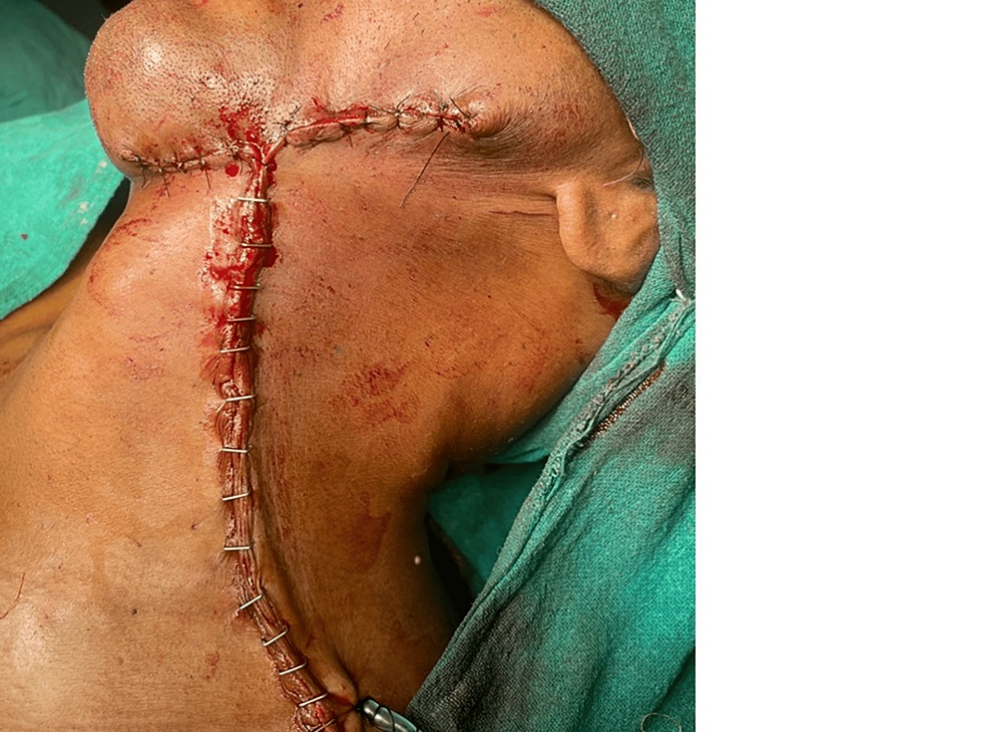
Post-operative image Primary closure was done on the left side after radical neck dissection.

The primary closure of the site was done after the placement of a negative suction drain as presented in Figure 6.

**Figure 7:**
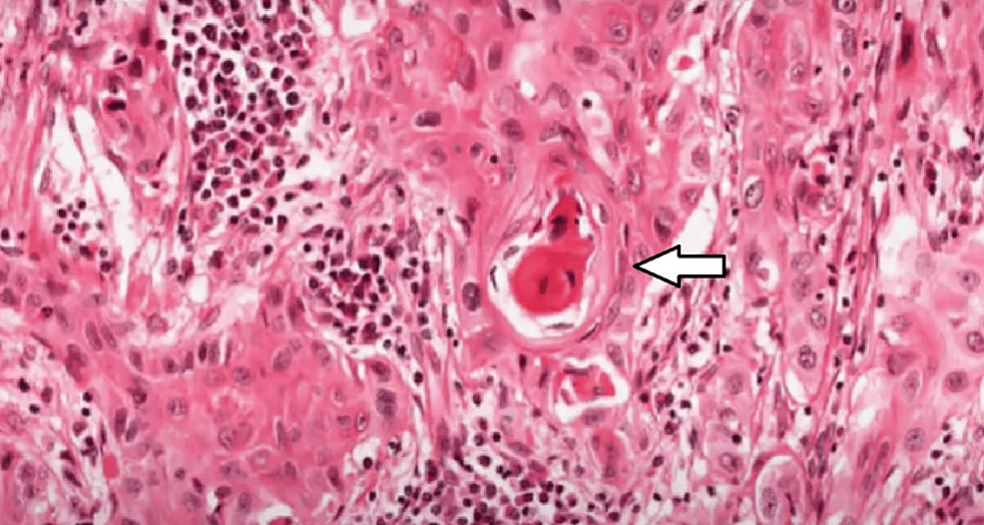
A histopathological slide of the resected specimen A histopathological slide of the resected specimen showing the presence of keratin pearl, suggestive of squamous cell carcinoma.

Histopathological examination of the resected specimen showed a unifocal, well-differentiated squamous cell carcinoma, seen in Figure 7, with a tumor depth of invasion of 12 mm. Twenty-two lymph nodes were identified, out of which eight were positive for malignancy with the presence of perineural invasion. For immediate post-operative care, the patient was given a standard injectable antibiotic regimen for 5 days and discharged after drain removal.

According to the American National Comprehensive Cancer Network (NCCN), postoperative radiotherapy (PORT) should ideally begin within 42 days of surgery, as recommended [4]. The patient was planned for adjuvant radiotherapy to the left face and neck with a dose of 60 gray in 30 fractions of intensity modulation radiotherapy with weekly cisplatin. After two months, the patient reported generalized weakness along with an ulcerative swelling in the left preauricular region. A fine needle aspiration cytology of the swelling was done, suggesting a recurrence of squamous cell carcinoma. The patient was then advised palliative chemotherapy and has remained on monthly follow-up.

## Discussion

Head and neck cancers of unknown primary (CUP) origin account for roughly 1-5% of all malignancies in this region. The initial assessment of a patient with a solitary neck mass should involve a comprehensive examination of their medical history and a thorough physical examination. In the South Asian Subcontinent, oral squamous cell carcinomas typically manifest as noticeable swellings and ulcerations. Consequently, it may pose a challenge for new clinicians when encountering cases where the primary lesion is not conspicuously evident. The identification of the primary site by modern functional imaging has significantly reduced the prevalence of true CUP, but these entities still exist [5]. Owing to this diagnostic conundrum, it is imperative to identify these entities for understanding their diagnostic workup and treatment planning. Cancers of unknown primary origin were previously included in the American Joint Committee on Cancer (AJCC) Tumour, node, and metastasis (TNM) staging 7th edition. A notable modification in the 8th edition of the TNM system, compared to previous editions, is the exclusion of the T0 category in locations other than the nasopharynx, Human papillomavirus-associated oropharyngeal cancer, and salivary gland cancers (which can be discerned through histology) [6].

During the initial clinical assessment, it's important to consider any symptoms that may indicate a possible primary site. Nasal obstruction, otitis media, or epistaxis could suggest a nasopharyngeal tumor, while dysarthria, dysphagia, or odynophagia may indicate an oropharyngeal tumor. Similarly, dysphonia could point to a laryngeal primary, while otalgia with normal ear examination may draw attention to the tonsils, base of the tongue, supraglottic area, or hypopharynx as potential primary sites. A thorough examination of the ear, nose, and throat is essential, including careful inspection and palpation of the oral cavity, tonsils, and tongue base. For submucosal cancers, a helpful technique for detecting the primary site clinically is to reexamine the patient for bleeding after initially palpating the tonsils and tongue base, along with retracting the tonsillar pillars. Additionally, a comprehensive examination of the head and neck region for both undiagnosed tumors and scars from previous cutaneous lesion treatments is important. 

Before specific imaging has been performed, the data obtained solely from visual examination and palpation of enlarged cervical lymph nodes is generally nonspecific. To assist in formulating a reasonable differential diagnosis, cervical lymphadenopathy can be classified into unilateral and bilateral lymphadenopathy with extranodal soft tissue lesions, and solitary massive lymphadenopathy. Systemic diseases like lymphoproliferative disorders, histiocytosis, post-transplant lymphoproliferative disease, and sarcoidosis can manifest with bilateral lymphadenopathy. Conditions characterized by a focal disease process triggering enlargement of regional lymph nodes cause unilateral lymphadenopathy. Examples include acute bacterial lymphadenitis, cat-scratch disease, mycobacterial lymphadenitis, malignant/metastatic lymphadenopathy, and foreign body-associated granulomatous lymphadenopathy. One of the most common presentations of extrapulmonary tuberculosis is a solitary neck mass. It manifests as firm, rubbery nodes that become matted as the disease progresses and may develop a draining fistula, with the overlying skin having a violaceous hue. When discussing solitary neck masses, it's important to note certain less common conditions such as Castleman's disease, also known as giant lymph node hyperplasia or angiofollicular lymph node hyperplasia. This disease can occur in both unicentric and multicentric forms. In the unicentric form, it presents as a large, solitary, densely homogeneously enhancing mass. These differential diagnoses can be considered prior to more invasive diagnostic methods.

The approach to CUP has evolved over the past decade, with the advent of sophisticated imaging and pathologic tests. Direct endoscopies, often referred to as triple endoscopies, tumour endoscopies, or panendoscopies, involve the thorough examination of the oral cavity, pharynx, larynx, along with the tracheobronchial tree, and esophagus, should be conducted in the diagnostic workup. The characteristics of neck metastases are crucial in evaluating these patients, with the location of the neck mass indicating potential primary tumor sites. Typically, the neck mass appears at level II, followed by level III, with bilateral involvement seen in a minority of cases. Bilateral metastases should raise suspicion for tumors in the nasopharynx, base of the tongue, hypopharynx, and midline structures. In certain cases, cervical lymphadenopathy unassociated with a visible carcinoma in the head and neck region may also be tied to a primary carcinoma of the submandibular gland. Physicians should assess for distant metastases where both upper and lower nodes are involved, which are more common in these patients than those with only the upper nodes. Classic imaging studies used in the workup of unknown primary carcinomas include computed tomography and magnetic resonance imaging. These are particularly useful when the primary tumor is suspected to be located outside the head and neck region. A CT scan from the skull base to the clavicle can assess the extent of cervical disease, its relationship to surrounding structures, extracapsular extension, retropharyngeal nodes, and contralateral neck status. MRI offers superior soft tissue resolution, especially for evaluating nasopharynx and oropharynx. However, these imaging modalities alone have limited sensitivity in identifying the primary tumor, ranging from 9% to 23%, which increases to 60% when suspicious radiologic findings prompt subsequent endoscopic biopsies [7]. Positron emission tomography (PET) and PET/CT fusion imaging can help detect small primaries not appreciated by anatomic imaging or physical examination. Studies have reported varying detection rates of primary tumors using PET/CT, ranging from 27% to 57%, with a sensitivity of 84% and a false-positive rate of 15% for oropharyngeal and lung primaries [8]. Despite PET's limitations, including false-positive/negative results, cost, radiation exposure, and limited availability, it remains a valuable tool in CUP diagnosis, guiding biopsy, disease extent determination, radiation therapy planning, and surveillance. The National Comprehensive Cancer Network (NCCN) guidelines recommend PET imaging for patients suspected of having an unknown primary cancer. If the primary site of the tumour is confirmed, a biopsy can be taken and immunohistochemistry can be done for cancer subtyping.

Even with a thorough investigation, a significant proportion of primary occult tumors, ranging from 50% to 70%, elude detection [9]. The prognosis for individuals with carcinoma of the cervical lymph nodes, where the primary tumor site is unknown, is heavily influenced by the stage of the neck disease. Treatment strategies typically focus on either the primary resection of the lymphatic mass or a combination of chemotherapy and radiotherapy, followed by resection once the tumor has diminished in size.

Yoshii et al. reported that neck dissection in 13 out of 15 (86.7%) cases of metastatic carcinoma of the cervical region of unknown primary was carried out, which showed an improvement in prognosis [10]. In a study conducted by Abu-Shama et al. in 2021, findings indicated that combining neck dissection with radiotherapy led to enhanced nodal control and improved progression-free survival in cases of head and neck cancers with an unknown primary [11]. This positive effect was observed across all nodal stages. Interestingly, advanced nodal presentation did not show compensatory benefits from neoadjuvant chemotherapy in the study. These results suggest a potential therapeutic advantage of combining neck dissection and radiotherapy in managing head and neck cancers of unknown primary origin, regardless of nodal stage, while highlighting the limited efficacy of neoadjuvant chemotherapy in addressing advanced nodal disease. 

Cervical lymphadenopathy unassociated with a visible carcinoma in the head and neck region may also be tied to a primary carcinoma of the submandibular gland [12]. In the case discussed in this report, it is intriguing to note that despite a radical neck dissection that had been performed to remove the tumor mass, which involved the removal of the submandibular gland, the patient experienced recurrence on the same side. 

## Conclusions

The challenges encountered in managing carcinomas of unknown primary origin should be addressed by a multidisciplinary team approach involving oncologists, radiologists, and pathologists. The inclusion of comprehensive imaging studies and molecular profiling of tumor tissue ensures proper guidance in treatment decisions. Increasing evidence suggests that CUP may actually consist of unrelated groups of site-specific tumors that share the characteristic of having a property of having a diminutive undetected primary. Additionally, there has been a shift from anatomically defined favorable subsets to pathologically defined subsets, where pathology holds more importance in the management of the disease. The utilization of new modalities, such as transoral robotic surgery and laser microsurgery in diagnosing the site of the unknown primary has become more prevalent. In the present case, we proceeded with radical neck dissection, and as the resected tissue was positive for perineural invasion, post-operative adjuvant radiotherapy was advised. The site of the primary lesion in the current patient remains to be identified, and swift treatment is essential in the event of its detection. As a result of the recurrence, the patient was advised palliative care and is under close follow-up, including PET-CT and MRI, at our department to ensure a timely response should the primary tumor be eventually detected.
